# Polyamino-Isoprenic Derivatives Block Intrinsic Resistance of *P*. *aeruginosa* to Doxycycline and Chloramphenicol *In Vitro*

**DOI:** 10.1371/journal.pone.0154490

**Published:** 2016-05-06

**Authors:** Diane Borselli, Aurélie Lieutaud, Hélène Thefenne, Eric Garnotel, Jean-Marie Pagès, Jean Michel Brunel, Jean-Michel Bolla

**Affiliations:** 1 Aix-Marseille Université, IRBA, TMCD2 UMR-MD1, Faculté de Médecine, 13385 Marseille, France; 2 Hôpital d'Instruction des Armées Alphonse-Laveran, 13013 Marseille, France; 3 Centre de Recherche en Cancérologie de Marseille (CRCM), CNRS, UMR7258, Institut Paoli Calmettes, Aix-Marseille Université, UM 105, Inserm, U1068, F-13009, Marseille, France; Centre National de la Recherche Scientifique, Aix-Marseille Université, FRANCE

## Abstract

Multidrug resistant bacteria have been a worldwide concern for decades. Though new molecules that effectively target Gram-positive bacteria are currently appearing on the market, a gap remains in the treatment of infections caused by Gram-negative bacteria. Therefore, new strategies must be developed against these pathogens. The aim of this study was to select an antibiotic for which a bacterium is naturally resistant and to use an escort molecule to restore susceptibility, similarly to the model of β-lactam/ β-lactamase inhibitors. High-content screening was performed on the reference strain PA01, allowing the selection of four polyamino-isoprenic compounds that acted synergistically with doxycycline. They were assayed against clinical isolates and Multi-Drug-Resistant strains. One of these compounds was able to decrease the MIC of doxycycline on the reference strain, efflux pump overproducers and clinical isolates of *P*. *aeruginosa*, to the susceptibility level. Similar results were obtained using chloramphenicol as the antibiotic. Membrane permeation assays and real-time efflux experiments were used to characterize the mechanism of doxycycline potentiation.

The results showed that the selected compound strongly decreases the efficiency of glucose-triggered efflux associated with a slight destabilization of the outer membrane. According to these data, targeting natural resistance may become an interesting way to combat MDR pathogens and could represent an alternative to already devised strategies.

## Introduction

With the current global crisis of antibiotic resistance, any strategy that could improve the therapeutic tools used in the fight against bacterial infections must be utilized [1]. National and International health agencies have successfully established a series of measures to encourage the control of the usage of drugs and the development of new molecules. Although significant progress has been made in the fight against Gram-positive infections in the last decade, a gap remains in the treatment of Gram-negative bacteria, particularly in the discovery of new active molecules [2, 3]. Effective Gram-positive targeting molecules are globally not very active against Gram-negative bacteria, though the same targets exist in both types of bacteria [4]. In the latter, the major problem of antibiotic entry and accumulation of the drug near the target has not yet been solved [5] This is due, in part, both to the low permeability of Gram-negative bacteria, which are surrounded by the outer membrane, that decreases the entry of compounds and to the constitutive expression of efflux pumps by these bacteria [6, 7]. Moreover, it is well documented that they are able to overproduce these efflux pumps in response to extra-cellular compounds, including drugs [8]. Thus, screenings based on an *in vitro* test for a particular target face the recurring problem of the permeability of Gram-negative bacteria [9].

Therefore, it may be interesting to first identify molecules effective on whole bacteria and then, if the test is positive, search for their mode of action. We used this approach to address the question of the natural resistance of Pseudomonas to doxycycline.

The aim of this study was to select an antibiotic for which a bacterium is naturally resistant and to use an escort molecule to restore susceptibility, similarly to the model of β-lactam/ β-lactamase inhibitors. This strategy could constitute an opportunity for an old neglected molecule to be rejuvenated by using an adjuvant to improve its action.

*Pseudomonas aeruginosa* represents a serious therapeutic challenge for both community-acquired and nosocomial infections. *P*. *aeruginosa* possesses an intrinsic high-level antibiotic resistance due a naturally low permeable outer membrane (1/100 of *E*. *coli* outer membrane). It makes the bacterium resistant to many classes of antibiotics [8]. Together with impermeability, the low susceptibility of *P*. *aeruginosa* to β-lactam antibiotics results from the overexpression of the intrinsic AmpC cephalosporinase, and the acquisition of extended-spectrum β-lactamases, of metallo-carbapenemases, and extended spectrum oxacillinases [10].

In addition, the inherent resistance of *P*. *aeruginosa* is, to a great extent, due to an active efflux of a broad spectrum of molecules. This efflux is mediated by three-component systems that belong to the Resistance Nodulation cell Division family of transporters (RND). MexAB-OprM and MexXY-OprM are the most studied [11–13] and the most prevalent in clinical isolates [14]. MexAB-OprM is constitutively expressed and confers the ability to export several classes of drugs [15]. The expression of MexXY-OprM contributes to both intrinsic and acquired resistance [16], and this resistance can be induced by antibiotics such as tetracyclines and macrolides [16].

In an on-going project dedicated to the synthesis of new biologically active molecules, we identified several polyamino-isoprenic derivatives that were able to decrease chloramphenicol- and nalidixic acid-resistance levels of multi-drug-resistant Enterobacterial strains [17].

In the present study, we describe the use of this chemical library to screen chemosensitizers that could lower the MIC of doxycycline on *P*. *aeruginosa* under the threshold of susceptibility. We also present data supporting the concept that polyamino-isoprenic compounds potentiate doxycycline’s action by circumventing the intrinsic resistance.

## Materials and Methods

### Bacterial strains and growth conditions

The *P*. *aeruginosa* strains used in this study are described in Table 1. In addition, a series of 20 clinical isolates of *P*. *aeruginosa* obtained from the Hôpital d’Instruction des Armées Laveran, HIA-Laveran (Marseille, France) were also used in this study (S1 Table). Strains were stored at -80°C in 15% (v/v) glycerol for cryo-protection. Bacteria were routinely maintained on Mueller-Hinton (MH) agar plates and grown in cation-adjusted MH broth (MHBII) at 37°C.

**Table 1:** Strains used in this study and their susceptibilities to doxycycline, chloramphenicol, and the compounds 1–4.

*Strains*	*Relevant phenotype*	*Source and reference*	Minimal Inhibitory Concentration mg/L (μM)*					
			*DOX*	*CHL*	*1*	*2*	*3*	*4*
**PA01**	wt	P. Plésiat [20]	32	256	> 85 (>250)	> 81 (>250)	25 (62.5)	49 (125)
**PT629**	Mex AB overproducer	P. Plésiat [21]	>32	512	> 85 (>250)	> 81 (>250)	25 (62.5)	49 (125)
**PA01 ERY**^**R**^	Mex CD overproducer	P. Plésiat [22]	16	256	> 85 (>250)	> 81 (>250)	25 (62.5)	25 (62.5)
**PA0-7H**	Mex EF overproducer	P. Plésiat [23]	>32	>1024	85 (250)	> 81 (>250)	25 (62.5)	49 (125)
**CMZ091**	Mex XY overproducer	P. Plésiat [24]	32	256	85 (250)	> 81 (>250)	25 (62.5)	49 (125)
**FB1**	Mex B deleted	P. Plésiat [25]	32	8	85 (250)	> 81 (>250)	25 (62.5)	49 (125)

DOX, doxycycline; CHL, chloramphenicol.

* Results are the mean of 3 independent experiments.

### Antibiotics and chemicals

The antibiotics doxycycline, polymyxin-B polymyxin-B nonapeptide (PMBN) and chloramphenicol and Phenylalanine-Arginine Beta-Naphthylamide (PAßN), carbonyl cyanide m-chlorophenylhydrazone (CCCP), cetyl trimethylammonium bromide (CTAB), 4-(2-hydroxyethyl)piperazine-1-ethanesulfonic acid (HEPES) and benzalkonium chloride were purchased from Sigma (St Quentin Fallavier, France). EDTA was from Fisher-Scientific (Quichy-le-château, France) The antibiotic imipenem was purchased from Pharmacopeia (USA). They were dissolved in water or dimethyl sulfoxide (DMSO) as indicated. The polyamino-isoprenic derivatives were described previously [18]. They were dissolved in DMSO and stored at -20°C until use.

### Antibiotic susceptibility testing

Assays were carried out in 96-well microtiter plates using a two-fold standard broth micro-dilution method, as previously described [19]. Experiments were performed on the BAC-SCREEN platform of the UMR-MD1, with a Freedom EVO 150 liquid handling system (Tecan, Lyon, France). The MIC of doxycycline, chloramphenicol and of the 4 compounds for each strain is given in Table 1.

### High-content screening

The polyamino-isoprenic compounds library [18] was screened against the *P*. *aeruginosa* PA01 reference strain (Table 1) in the presence of 4 mg/L doxycycline that corresponds to the concentration under the threshold of resistance according to the CLSI guidelines, in order to evaluate if one of the compound is able to decrease the intrinsic resistance of *P*. *aeruginosa*. For further experiments we decided to reduce the concentration of doxycycline at 2mg/L to increase the stringency of the tests. Indeed, this concentration corresponds to the threshold of the susceptibility according to the CLSI. The screening was carried out in 96-well microtiter plates using MHBII, in a final volume of 200 μL. The concentration of library compounds was 10 μM, with a final DMSO concentration of 2.5%. The bacterial inoculum of 5×10^5^ CFU/mL was prepared from an overnight culture. In addition to the polyamino-isoprenic compounds, each plate contained PAßN, CCCP, CTAB, polymyxin-B and benzalkonium chloride as control molecules. Plates were incubated at 37°C under aerobic conditions for 18 hours. Plates were read at 600 nm on an Infinite M200 Pro plate reader (Tecan). Results are summarized in S1 Fig.

### Comparison of efficiency between compounds and PAßN

Serial dilutions of each compound (from 270 μM to 0.95 μM for PAßN and from 135 μM to 0.06 μM for 3) were prepared in microplates. Doxycycline (2 mg/L) was then added to each well before inoculation of bacteria (5x10^5^ cfu/mL). Optical density (600 nm) was measured after 18 hours at 37°C. Only the relevant part of the graphs are shown. Experiments were performed in triplicate.

### Checkerboard assay

Serial two-fold dilutions of antimicrobial agents were mixed together in a microtiter plate. The range of concentration of each drug was twice the MIC, down to the eleventh serial two-fold dilutions below this amount. Doxycycline was serially diluted along the X-axis, and the polyamino-isoprenic compound or PAßN was diluted along the Y-axis as indicated. Each well was inoculated with a bacterial inoculum of 5 x 10^5^ CFU/ml, and the plates were incubated at 37°C for 18 h under aerobic conditions. Each plate also contained a row and a column in which a serial dilution of polyamino-isoprenic compound or doxycycline, respectively, was present alone to determine the MIC. The FIC index was calculated as follows: FIC index = FIC A + FIC B, where FIC A is the MIC of drug A in the combination/MIC of drug A alone and FIC B is the MIC of drug B in the combination/MIC of drug B alone. The combination was considered synergistic when the FIC index is ≤0.5, and antagonistic when the FIC index is > 4 [26]. Results are described in S2 Table.

### Effect of combined antibiotics–polyamino-isoprenic derivatives

MH agar plates were prepared, containing 2 mg/L doxycycline and 2 mg/L doxycycline in combination with compounds at a concentration of 10 μM. Bacteria, 5 μL of a suspension of each clinical isolate adjusted to 5 x 10^5^ colony-forming units per ml were spotted on each plate and growth was monitored after a 18 h period of incubation at 37°C. The isolates that repeatedly grown on the plates containing doxycycline alone and compound alone but that did not grow on both doxycycline and compound, were identified as susceptible of the combination.

In a second experiment, 5 μL of logarithmic dilutions of an overnight culture of the 6 strains described in Table 1 were spotted on the MH agar plates prepared as above. An additional assay was also performed with these 6 strains using chloramphenicol as antibiotic instead of doxycycline.

### Outer membrane permeation assay

An overnight culture of PA01 was diluted 100-fold into 10 ml MHII broth containing 0.01 μg/ml of imipenem to induce a higher level of β-lactamases. After reaching an OD600 nm of 0.5, cells were recovered by centrifugation (4,000 X g for 20 min) and washed twice in 20 mM potassium phosphate buffer (pH 7.2) supplemented with 1 mM MgCl_2_ (PPB) at an OD 600 nm of 0.5. 50 μl of each compound was added to 100 μl of the cell suspension, yielding final concentrations ranging from 125 μM to 15.6 μM. Then, 50 μl of nitrocefin was added to obtain a final concentration of 50 μg/ml. Absorbance at 490 nm was monitored by spectrophotometry using an Infinite M200 microplate reader (Tecan) over 60 min. Experiments were performed in triplicate. For each compound, the efficacy of permeation was determined using the slope in the linear range, relatively to the slope obtained with 250 μM polymyxin-B.

### Real-time efflux assay

The experiments were performed as previously described [17] with slight modifications. For each strain, 20 mL of MHII was inoculated with a single colony from a fresh plate and grown at 37°C to the stationary phase. The cells were then recovered by centrifugation (4,000 X g for 20 min) and washed once in 20 mM PPB. They were then loaded over night with the dye 1,2’-dinaphthylamine (TCI-Europe SA, Zwijndrecht, Belgium) at a final concentration of 32 μM, in the presence of 5 μM CCCP. The cell suspension was then recovered by centrifugation (4,000 X g for 20 min) and re-suspended in the same volume of PPB and adjusted to an OD600 nm of 0.5. In a 96-well Greiner black microplate (Greiner, Courtaboeuf, France), a gradient of inhibitor (when used) was prepared in PPB, and bacteria were added (100 μL per well). The fluorescence of the cell suspension, was monitored each 22 sec during 1200 sec on an Infinite M200 microplate reader (Tecan; excitation wavelength 370 nm and emission wavelength 420 nm). In order to trigger the transport, glucose (50 mM final concentration) is added at 200 sec. The maximum efficacy of the efflux is the difference between the value obtained without and with glucose addition after 1200 sec. For each compound, the efficiency of efflux inhibition is calculated as the difference between the maximum efficacy of transport and the residual transport obtained at various concentrations of the compound.

### Cytotoxicity assessment

CHO-K1 cells (ATCC-LGC Standards Sarl (Molsheim, France) and human fibroblasts (Clinisciences, Paris, France) were maintained in McCoy’s 5A and DMEM media, respectively, supplemented with 10% bovine calf serum, 2 mM glutamine, and 100 U/mL/10 μg/mL penicillin/streptomycin. They were incubated at 37°C in a humidified atmosphere containing 5% CO_2_. The cell lines were seeded in 96-well plates and incubated overnight. The cytotoxic effects of compounds were assessed by the colorimetric WST-1 cell proliferation assay as previously described [17]. Briefly, a range of compound concentrations from 30 μM to 1200 μM was added to triplicate cultures, and cells were incubated at 37°C for 24 h. At the end of the incubation period, cultures were washed three times with phosphate buffer saline (PBS) and incubated in fresh culture medium containing 10% WST-1 for an additional 30 min. Cell viability was evaluated by measuring the WST-1 absorbance at 450 nm in a microplate spectrophotometer MRX1 II (Dynex technologies, Chantilly, VA, USA). The Inhibitory Concentration 50% (IC_50_) was chosen to evaluate the cytotoxicity of compounds. IC_50_ was defined as the concentration of a compound that induced a 50% decrease in viable cells. Doxorubicin was used as a positive control.

### Statistical analysis

Data were analyzed using the student t-test analysis for differences between two groups, and findings were expressed as mean + SD. All assays included 2 replicates and were repeated in at least 2 independent experiments. A p value < 0.05 was considered to be statistically significant.

## Results

### Selection of polyamino-isoprenic compounds

**Fig 1:**
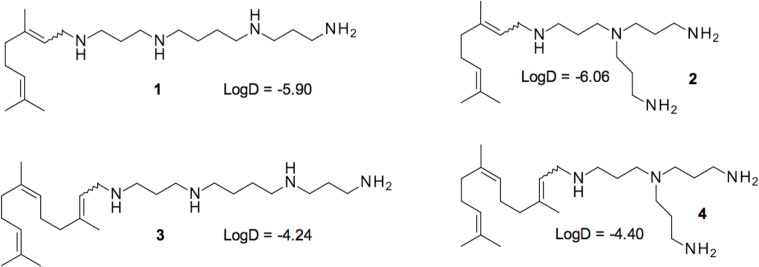
Structure of polyamino-isoprenic derivatives 1–4 identified in this study. LogD have been determined by using chemical simulation software Marvin Sketch 5.11.3.

A series of 60 compounds, which were described [18], were tested at a concentration of 10 μM in combination with a sub-inhibitory concentration of doxycycline against the reference strain of *P*. *aeruginosa* PA01. In parallel, we evaluated the intrinsic antimicrobial activity of these compounds alone to allow the selection of only doxycycline adjuvants. The results are summarized in S1 Fig. Under these conditions, three compounds, **2**–**4** (Fig 1), were able to decrease the MIC of PA01 for doxycycline below the threshold of resistance (4 mg/L), [27]. Interestingly, these three compounds share similar structures with however, very distinct LogD values (Fig 1). Compounds **2** and **4** share the same polyamine and only differ in their isoprenyl moiety. Conversely, **3** and **4** share the same isoprenyl group and differ in their polyamines. In the subsequent experiments, we also considered compound **1**, which has the same isoprenyl group as **2** and the same polyamine as **3** but did not show any significant synergy.

### Cytotoxicity of the selected compounds

The cytotoxicity on Chinese Hamster Ovary cells and human normal fibroblasts, was determined using the metabolic WST1 assay for the four compounds (Table 2). Compound **1** appeared to be the least cytotoxic on both cell lines, with values ranging from 20 times for the CHO to 10 times for human fibroblasts compared with the positive control, the doxorubicin. Compounds **2** and **3** showed similar values that were lower than those of **1** but still clearly above those of doxorubicin for the CHO cell line. Compared with **2** and **3**, compound **4** exhibited the same toxicity on CHO cells and lower toxicity on human fibroblasts.

**Table 2:** WST1 cytotoxicity test of the compounds.

Compound	CHO	Fibroblast
	IC_50_ (μM)*	IC_50_ (μM)*
**1**	808 +/- 68.1	138 +/- 18.6
**2**	235.5 +/- 28.9	Nd**
**3**	320.5 +/- 41.2	Nd**
**4**	363 +/- 20.3	136 +/- 7.9
**Doxorubicin**	48.3 +/- 6.8	12.4 +/- 2.1

* mean +/- Standard deviations, data are obtained from 2 independent experiments

** Not determined.

### Efficacy comparison of the selected compounds

PA01 is a well-studied reference strain of *P*. *aeruginosa*, however to extend our study we considered strains recently isolated from hospitalized patients [21–25]. Thus, we decided to test this combination of doxycycline (2mg/L) with polyamino-isoprenic compounds (10 μM) on twenty clinical isolates recently isolated from the HIA-Laveran (see M&M section and S1 Table).

**Fig 2:**
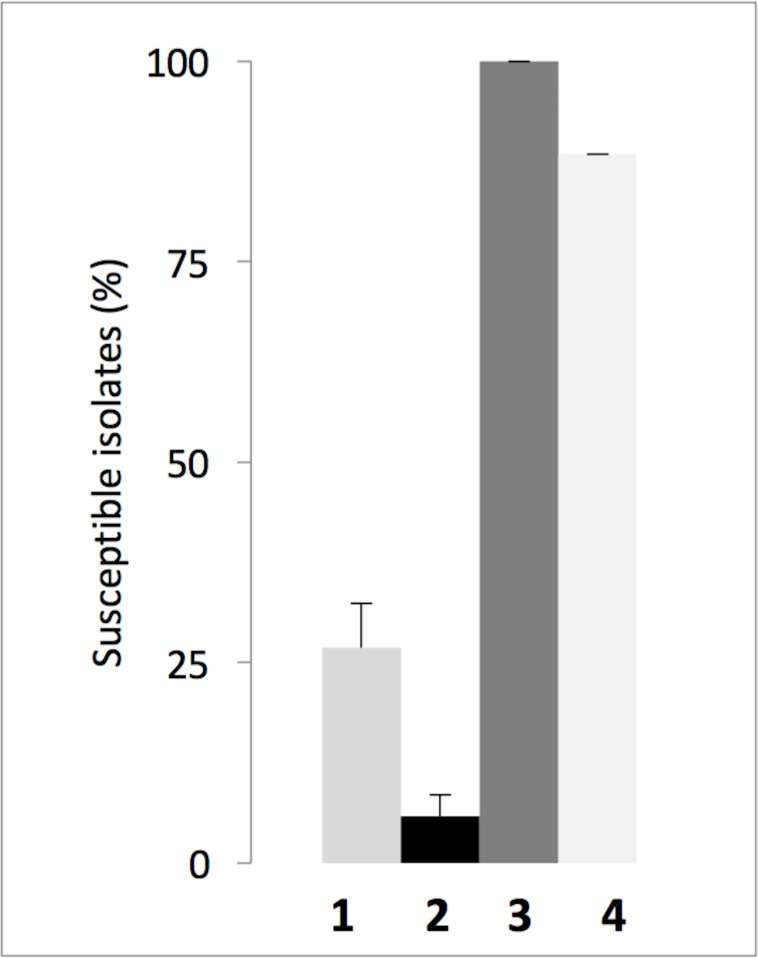
Susceptibility of clinical isolates of *P*. *aeruginosa* to treatment with doxycycline in combination with various polyamino-isoprenic derivatives. The percentage of susceptible isolates to doxycycline (2 mg/L) supplemented with one of the compounds (10 μM) was determined for each combination. grey bar, black bar, dark grey bar, and white filled bar, correspond to compounds **1**, **2**, **3** and **4** respectively. Error bars represent standard deviations of 3 independent experiments.

Parent derivatives **1** and **2** showed only a slight efficacy, restoring doxycycline susceptibility in only 27% and 6% of the isolates, respectively. Compounds **4** restored the doxycycline susceptibility of 88% of the isolates (Fig 2), whereas compound **3** led to a total susceptibility of all the considered isolates with a MIC ratio of 32 (S1 Table). We concluded that compound **3** was the best compound and suitable for a more detailed study. The same experiment was performed using chloramphenicol (4 mg/L) in combination with **3** (10 μM final concentration). Amongst the 20 clinical isolates, the growth of 15 (75%) was impaired, while 5 (25%) were resistant (data not shown). These experiments were performed to study the effect of our compounds on the natural resistance of *P*. *aeruginosa*. In addition, we were wondering if compound **3** could also increase susceptibility to other antibiotic families including ß-Lactams, fluoroquinolones and aminoglycosides, that are representative of the main classes of anti-pseudomonal agents. Experiments were performed with the ß-lactams ceftazidime and ticarcillin and ciprofloxacin and amikacin. Results are summarized in S3 Table. We found that, neither compound **3** nor PAßN (used as a control) showed significant synergy with ceftazidime on all the strains tested. A slight effect however, was observed in the combination of **3** with ticarcillin on all the strains. No synergistic effect of **3** with ciprofloxacin was observed with the exception of PA0-7H (that overproduces MexEF) where a 4 times decrease of MIC was measured. Conversely, a surprising increase of the MIC of amikacin was caused by **3** on every strains, this could be attributed to the deleterious effect of **3** on the proton gradient (see below), indeed the influx of aminoglycosides into bacteria is depending on the proton gradient through the inner membrane [28]. To better characterize compound **3**, we first searched for the minimum concentration of compound that allowed killing of bacteria by doxycycline, and PAßN was used as the reference molecule.

#### Determination of the minimum efficient concentration of compound 3

PAβN is the most characterized efflux pump inhibitor (EPI) in *P*. *aeruginosa*. Initially considered a promising alternative to prevent antibiotic resistance, its use as a prospective drug was discontinued because of its toxicity [29]. Nevertheless, it remains a useful tool for studying resistance of Gram-negative bacteria. The efficiency of compound **3** was further compared with that of PAßN.

**Fig 3:**
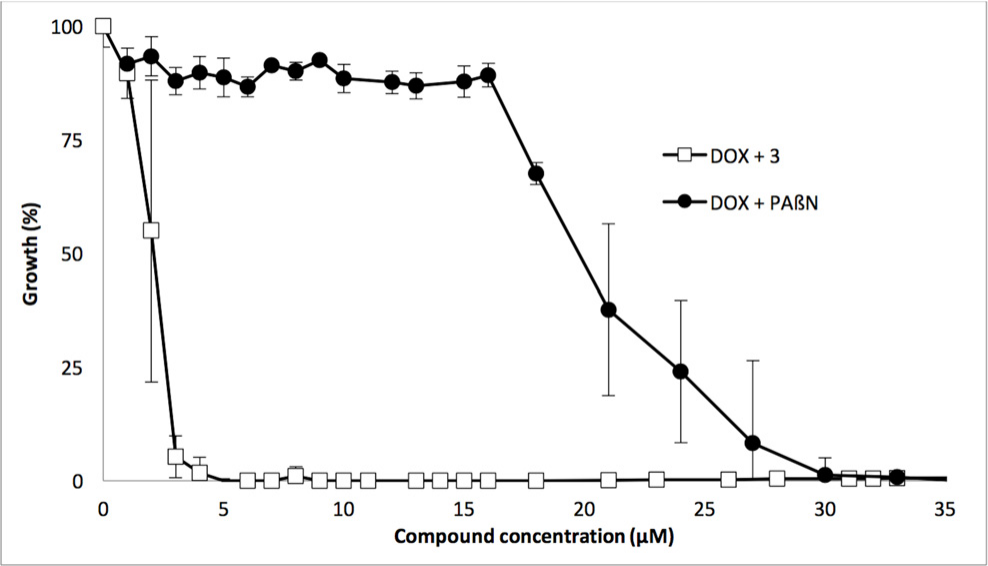
Comparison of the synergistic activity of PAßN and compound 3 with doxycycline. Bacterial growth corresponding to each concentration of compound was calculated as a percentage of bacterial growth under the same conditions without the addition of compound. Black filled circles, PAßN; open squares, Compound **3**. Error bars represent standard deviations of 3 independent experiments.

First, we performed a checkerboard assay to analyze the synergy between doxycycline and the polyamino-isoprenic compound and between doxycycline and PAβN. For both, compound **3** and PAβN we observed a synergistic inhibition of growth when combined with doxycycline with a FIC Index of 0.09 (S2 Table). Nevertheless, to more precisely refine these results, we fixed the concentration of interest of doxycycline at 2 mg/L (see above) and tested a gradient of concentrations for PAßN and **3**. PAßN is generally used at a concentration greater than 10 μM to inhibit the resistance of *P*. *aeruginosa* with respect to various antibiotics [30], and, to our knowledge, no data have been reported in the literature on its use in combination with doxycycline. The percentage of growth inhibition was monitored as a function of compound concentration, and the results are presented in Fig 3. Under these conditions, it clearly appears that at least a 30 μM (15.6 mg/L) concentration of PAßN is necessary to efficiently inhibit the growth of *P*. *aeruginosa* in the presence of sub-inhibitory concentration of doxycycline, which is in agreement with the efficacy of PAßN to restore fluoroquinolone susceptibility of *Pseudomonas* [30]. Conversely, a concentration of approximately 3 μM (1.22 mg/L) of **3** was sufficient to significantly inhibit PA01 growth (Graphically determined on Fig 3) and confirmed with the checkerboard assay (S2 Table). According to these data, **3** appears to be a stronger adjuvant of doxycycline than does PAßN *in vitro*. This first set of assays was performed on the reference strain PA01, which exhibits only a natural resistance towards doxycycline.

### Action of compound 3 on efflux pumps

**Fig 4:**
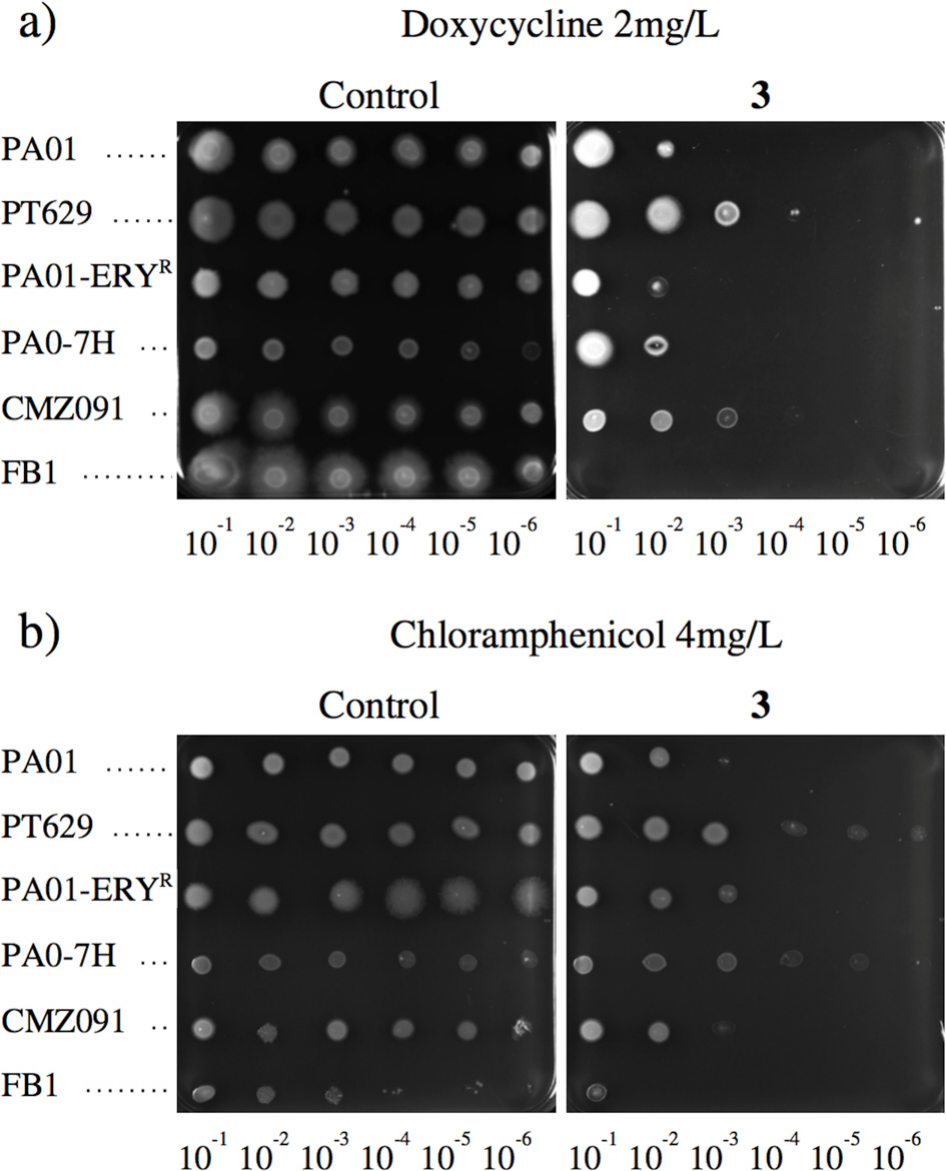
Compound 3 activity against *P*. *aeruginosa* strains over-expressing efflux pumps. The ability of bacteria to grow under various conditions was assessed in solid media containing, in a: 2 mg/L doxycycline (left) or a combination of 2 mg/L doxycycline and 10 μM compound **3** (right) and in b: 4 mg/L chloramphenicol (left) or a combination of 4 mg/L chloramphenicol and 10 μM compound **3** (right). 5 μL of logarithmic dilutions of an overnight culture of each strain were spotted on the plates. From top to bottom, PA01, PT629, PA01-ERY^R^, PA0-7H, CMZ091 and FB1.

We hypothesized that if compound **3** targets the intrinsic resistance, it should also be effective on strains with acquired resistance, such as through the efflux mechanisms which are well known to contribute to the MDR phenotype [31]. The strains overproducing the major efflux pumps of *P*. *aeruginosa* used in this experiment are listed in Table 1. All of the strains grew well on the agar medium containing 2 μg/mL of doxycycline (Fig 4). In contrast, in the presence of both, doxycycline and **3**, PA01 or strains overproducing either MexCD or MexEF cells growth was observed when a dilution of 10^−2^ was plated on MH. Cells overproducing either MexAB or MexXY grew at a higher dilution (10^−4^). Nevertheless, in all cases, no growth was observed above a dilution of 10^−5^. Moreover, the cells from the Δ*mexB* strain did not grow at any of the dilutions, in accordance with the contribution of MexAB-OprM to cycline resistance [15]. According to these data we wondered if compound **3** could also restore or at least increase susceptibility of *Pseudomonas* to other antibiotics. We thus decided to test chloramphenicol because *P*. *aeruginosa* also exhibit a high level of intrinsic resistance to this drug (Table 1). Similar results were obtained at least for strains PA01 and CMZ091 and to a lesser extent for PA01-ERY^r^. For PT629 and PA0-7H a slight effect was also observed, (Fig 4b). These data suggest that the selected compound was able to efficiently affect resistance to different classes of antibiotic.

Taken together, these results demonstrate that the combination of doxycycline and **3** increases susceptibility of all the strains of *P*. *aeruginosa* overproducing the major efflux pumps, suggesting a possible inhibition of this resistance mechanism. According to these data and to the hyper-susceptibility of the *mexB* deficient mutant observed in Fig 4, we hypothesized that **3** could inhibit the efflux pumps of *P*. *aeruginosa*. However, at this time, we could not exclude the possibility that increased permeability might also explain the results obtained.

**Fig 5:**
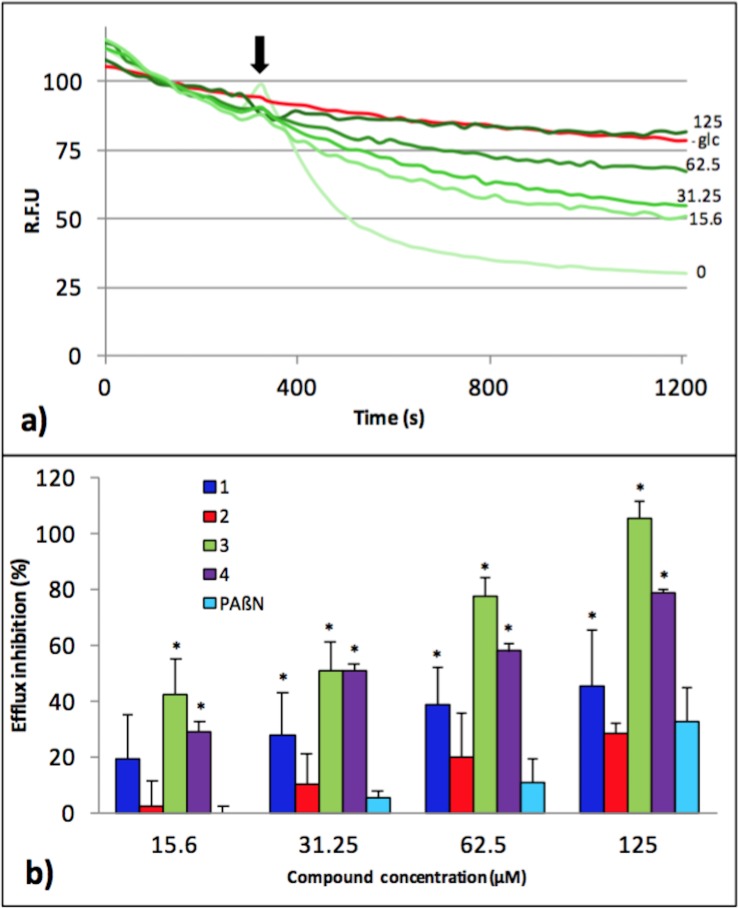
Inhibition of the efflux of the 1,2’ dinaphthylamine dye by the compound 3. Efflux was triggered after 200 s by the addition of 50 mM glucose (arrow). The intensity of fluorescence emission for 1,2’ dinaphthylamine is given in relative fluorescence units (RFU). (a) Concentration-dependent inhibition of 1,2’ dinaphthylamine efflux by compound **3** after glucose addition (arrow). Compound concentration (μM) is indicated in front of each green curve; as a control, an experiment without addition of compound and glucose (-glc, red curve) was also included; (b) percent of efflux inhibition obtained for each compound and PAßN as indicated. Error bars represent standard deviations of 2 independent experiments. * indicates statistically significant difference in values (p < 0.05) compared to PAßN.

To better understand whether these compounds were actually able to inhibit efflux, we developed a real-time efflux assay for *Pseudomonas* that was previously described for *Enterobacteriaceae* [17]. As shown in Fig 5A, the strain PA01 was able to efficiently expel 75% of the preloaded dye, immediately after glucose addition, thus demonstrating a strong activity of dye efflux in this strain. When compound **3** was added before glucose addition, a concentration-dependent inhibition of efflux was observed, and a concentration of 125 μM of **3** completely abolished the transportation of dye (Fig 5). The same experiments were performed with all the four compounds, in the same range of concentrations, and the results are summarized in Fig 5B as a percentage of efflux inhibition relative to the compound's concentration. For each compound tested, including PAßN, efflux is inhibited in a dose-dependent manner. One can mention that compound **3** is the most efficient, which is consistent with the data obtained in the susceptibility assays described in Fig 2.

**Fig 6:**
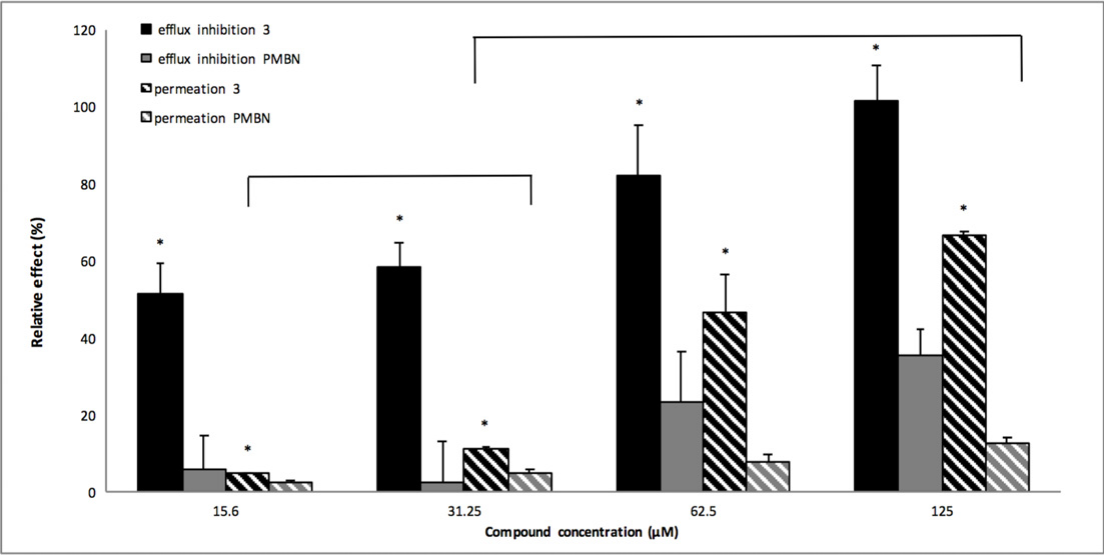
Membrane permeation assays. The effect on the PAO1 strain of compound **3** (black-filled and black-hatched bars) and PMBN (grey-filled and grey-hatched bars) were compared at concentrations ranging from 15.6 μM to 125 μM, as indicated. The relative effect corresponds either to efflux inhibition (filled-bars) or to outer-membrane permeation (hatched-bars). Error bars represent standard deviations of 2 independent experiments. * indicates statistically significant (p ≤ 0.05) differences of relative effect (efflux inhibition/outer membrane permeation) for compound **3** treatment compared to the respective PMBN treatment. Brackets indicate statistically comparable permeation effects.

Recent studies have shown that the mode of action of PAßN includes not only efflux inhibition but also outer membrane destabilization [32, 33]. Considering that our compounds are polyamine derivatives and that polyamines are known to disrupt membranes [34], one cannot exclude that the inhibition of glucose-triggered efflux of the dye described in Fig 5 could result, at least in part, from the disruption of the outer membrane of *P*. *aeruginosa* and consequently from the destabilization of the interactions between the partners of the RND complex that were shown necessary to ensure transport *in vitro* [35]. To evaluate the potential ability of **3** to permeabilize the outer membrane, we monitored the hydrolysis rate of the chromogenic β-lactam, nitrocefin, by the cells of *P*. *aeruginosa* PA01. The polymyxin-B derivative PMBN was previously described as having a strong permeation activity on the outer membrane of Gram-negative bacteria [24]. Therefore, we compared **3** with PMBN in permeation and efflux inhibition assays. We observed (Fig 6), that the permeation efficacy of **3** at 15.6μM was significantly identical to 31.25μM of PMBN (as indicated with bracket on Fig 6; p value ≤ 0.05). At the same concentrations, **3** already showed a fifty percent efflux inhibition, while PMBN was quite inefficient. The same analysis can be drawn for the respective concentrations of 31.25μM of compound **3** and 125μM of PMBN. In this case one can observe a 10 percent permeation activity for both, and a 60 percent efflux inhibition for **3** compared to 30 percent for PMBN (Fig 6). In addition, we investigated the activity of **3** on PA01 using a fluorescence assay that monitors cytoplasmic membrane depolarization [36]. The compound **3** dissipated the proton motive force (S3 Fig). This result may explain the antagonism of **3** with the aminoglycoside antibiotic amikacin (S3 Table), which requires the component of the transmembrane electrochemical gradient for cell penetration [28].

## Discussion

Here, we describe the study of new chemosensitizers that are able to increase the susceptibility to doxycycline of intrinsically resistant *P*. *aeruginosa*. *P*. *aeruginosa* is one of the most prevalent human pathogens and is known to be difficult to eradicate using the current therapeutic strategies. This is due to its natural antibiotic resistance and its strong ability to acquire additional mechanisms of resistance. In the present study, we aimed to identify a combination drug and new molecule that could be effective on both wild type and clinical strains. We used doxycycline as the antibiotic of choice because it is not used as a human therapeutic to treat infections with *P*. *aeruginosa* due to its low susceptibility. A preliminary study had previously demonstrated that polyamino-geranic compounds, in combination with doxycycline, were able to restore susceptibility of *Enterobacteriaceae* to antibiotics [17].

A prerequisite screening of original molecules was performed in a high-content procedure on the reference strain PA01 using a 10 μM concentration of our bioactive compounds [36] and a concentration of 4 mg/L of doxycycline. Although several compounds showed activity by decreasing bacterial growth in combination with doxycycline, we focused our studies on compound **3**, which completely abolished bacterial growth. Using the checkerboard assay, the FIC index of the compound **3**-doxycycline combination was determined to be 0.09, demonstrating a strong synergy between the molecules. Additionally, we observed that derivative **3** strongly decreased the resistance of MDR strains to both doxycycline and chloramphenicol, which belong to different antibiotic families. This observation suggests that this polyamino-isoprenic molecule may directly impair the natural resistance mechanisms of *P*. *aeruginosa*. This compound is a polyamino-farnesyl molecule and possesses a tri-isoprenyl group that could help the molecule pass through the outer membrane of *P*. *aeruginosa*, which is known to be strongly impermeable due to the presence of a highly hydrophobic lipid bilayer, in addition to a positively charged spermine moiety that could interact with the negatively charged outer membrane of the bacteria. In comparison with PAßN, the most studied chemosensitizer of *P*. *aeruginosa* toward antibiotics [30], compound **3** appeared to be approximately 10 times more potent (Fig 2). Interestingly, the same type of assay performed against *Enterobacteriaceae* identified not derivative **3** but compound **2** as the most potent (data not shown). These two compounds differ in both the size of the isoprenyl moiety and the structure of the involved amino group (Fig 1). Taking into consideration the LogD parameter, which reflects the true behavior and bioavailability of an ionizable compound in a solution at a given pH, a significant correlation with anti-pseudomonal activity was observed. Indeed, derivative **3** displaying the highest LogD values (e.g., the most hydrophobic derivative) also possesses higher activity than compounds **1**, **2**, **4**. Moreover, it clearly appears (S2 Fig) that derivative **3** is ionized as tri or tetra protonated species at a physiological pH enhancing its ability to interact with the negative charges present at the surface of the outer membrane of bacteria.

When tested in association with doxycycline against a series of clinical isolates, the high potency of derivative **3** was confirmed, with all of the clinical isolates being killed.

To determine the cytotoxicity of these compounds, they were tested on Chinese hamster ovary (CHO) cells and exhibited an IC50 of over 100 μM, which is at least 10 times the concentration used in this study (Table 2). However, we also observed a higher toxicity for human fibroblasts of compounds **1** and **4**. Consequently, we cannot exclude the same increase for compounds **2** and **3**. In addition, in all of the assays performed, we observed that the compounds are less cytotoxic than the control molecule doxorubicin.

To better understand the mode of action of compounds, we performed real-time efflux assays. We observed a dose response effect for each compound, with compound **3** being the most efficient at inhibiting the 1,2’-DNA efflux (Figs 5 and 6). As described in Fig 5, a relatively high concentration of **3** (31.25 μM) is needed to inhibit 50% of 1,2’DNA efflux. This might result from the setting up of a real-time assay that requires a high number of bacteria (2.5x10^8^ cfu/mL) compared to MIC determination (5x10^5^ cfu/mL) combined to an immediate observable effect. This compound was further assayed for its ability to permeabilize the outer membrane of *P*. *aeruginosa*. Compared to PMBN, compound **3** partially destabilizes the outer membrane, and strongly inhibits efflux pumps, (Fig 6). Taken together, these data strongly suggested that although **3** destabilizes the outer membrane, this effect cannot completely explain the efflux inhibition observed. In addition, we observed that **3** induced a significant depolarization of the inner membrane of PA01 (S3 Table). Taken together, according to the proton gradient dependence of the RND-efflux pumps, these data suggest that **3** inhibits efflux in *P*. *aeruginosa* by depleting the energy of the pumps.

Nowadays, there is no doubt that the emergence and the dissemination of bacterial resistance towards a drug is directly related to its rate of use [37]. The continuous development of drug resistance mechanisms linked to drug usage is also exemplified by the observed decrease in chloramphenicol resistance in countries where its use is strongly controlled [38]. Here, we have determined that an association between doxycycline and a polyamino-isoprenic compound killed *P*. *aeruginosa* reference strain, efflux overproducers and clinical isolates. To our knowledge, it is the first report of such an activity and may open a new field of investigation, associating pharmaco-chemistry and microbiology to develop molecules with better efficiency on bacteria and decreased toxicity. Since it is necessary to continuously develop alternate solutions to circumvent bacterial adaptation and due to the paucity of new drugs in the pharmaceutical company pipelines [39], our strategy could constitute an opportunity for neglected molecules to be rejuvenated by using “escort molecules” to improve their action. Our study paves the way for this type of new original antibacterial strategies.

## Supporting Information

S1 Fig Results of the screening procedure. (*) the compounds NV730, NV731, NV716 and NV720 are the compounds **1, 2, 3, 4** selected in this study respectively. (**) The concentration of 10 μM used in this study is over the MIC of *Pseudomonas aeruginosa* strain PA01 for polymyxin-B.
(TIFF)

S2 Fig Determination of LogD and protonated species for derivative 3. All LogD and protonated species involved for derivative 3 have been determined by using chemical simulation software Marvin Sketch 5.11.3.
(PDF)

S3 Fig Inner membrane depolarization by 3. The membrane potential disruption was followed by monitoring Disc_3_(5) fluorescence.
(PDF)

S1 Table Description of the clinical isolates. The antibiotic resistance of each isolate for commonly used antibiotics and the MIC for doxycycline are indicated.
(PDF)

S2 Table Results of the checkerboard assay. For each concentration of the combination between, (a) doxycycline and compound **3**; (b) doxycycline and PAßN, the FIC index is indicated.
(PDF)

S3 Table Results of the synergy assays of compound 3 with representative anti-pseudomonal agents. (PDF)
