# Supplementary figures and images for: Polyamino-Isoprenic Derivatives Block Intrinsic Resistance of P. aeruginosa to Doxycycline and Chloramphenicol In Vitro
Source: PLoS One. 2016 May 6;11(5):e0154490. doi: 10.1371/journal.pone.0154490 (PMC4859512; doi:10.1371/journal.pone.0154490)

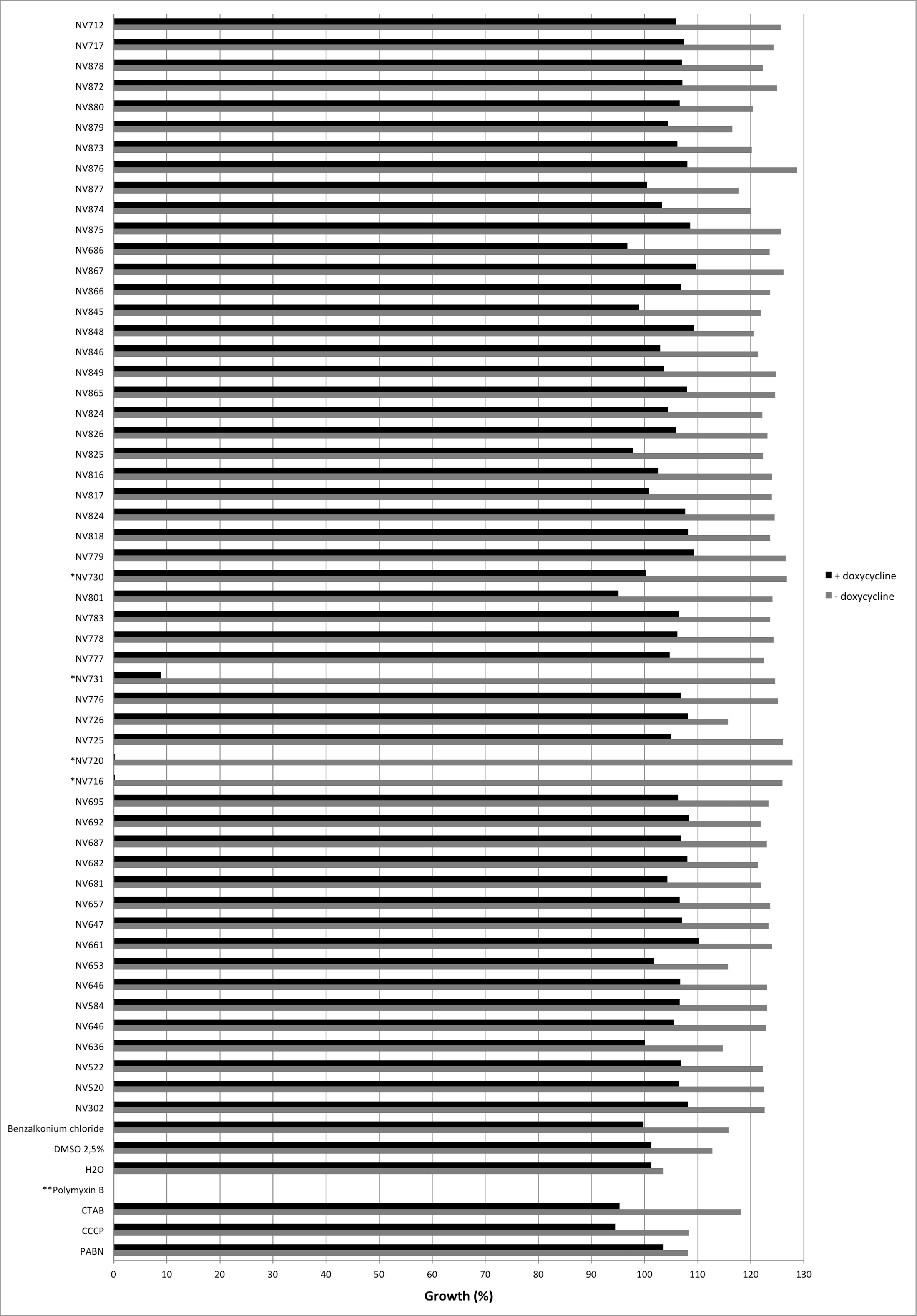

Supplement: S1 Fig — (*) the compounds NV730, NV731, NV716 and NV720 are the compounds 1, 2, 3, 4 selected in this study respectively. (**) The concentration of 10 μM used in this study is over the MIC of Pseudomonas aeruginosa strain PA01 for polymyxin-B. (TIFF) [file pone.0154490.s001.tiff]
