# Supplementary material for: Polyamino-Isoprenic Derivatives Block Intrinsic Resistance of P. aeruginosa to Doxycycline and Chloramphenicol In Vitro
Source: PLoS One. 2016 May 6;11(5):e0154490. doi: 10.1371/journal.pone.0154490 (PMC4859512; doi:10.1371/journal.pone.0154490)

Chemical parameters

a) Estimation of protonated species encountered at various pH for compound **3**.

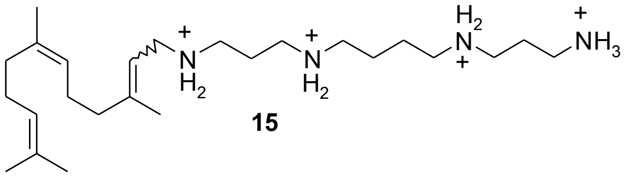

15

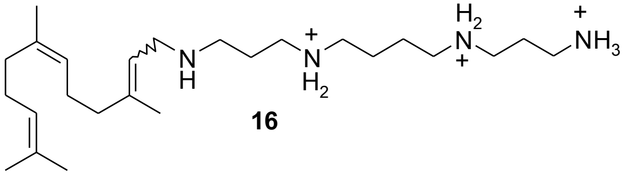

16

At pH 7.0 species **16** in 90.7% and species **15** in 4.99%

At pH 7.2 species **16** at 85.89% and species **15** in 7.49%

b) Identification of the major species according to pH theorical values.

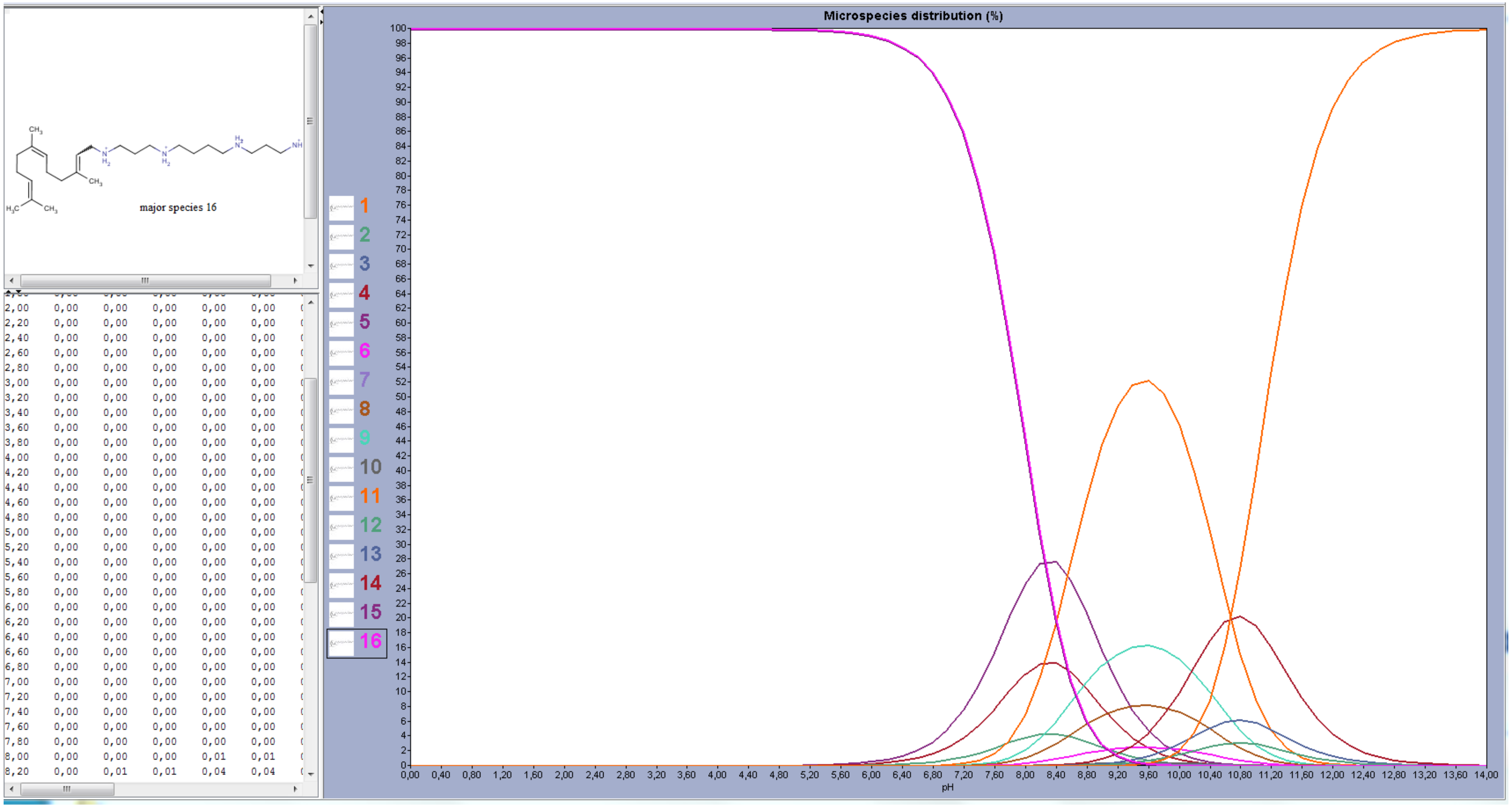

Supplement: S2 Fig — All LogD and protonated species involved for derivative 3 have been determined by using chemical simulation software Marvin Sketch 5.11.3. (PDF) [file pone.0154490.s002.pdf]
