# Supplementary material for: Polyamino-Isoprenic Derivatives Block Intrinsic Resistance of P. aeruginosa to Doxycycline and Chloramphenicol In Vitro
Source: PLoS One. 2016 May 6;11(5):e0154490. doi: 10.1371/journal.pone.0154490 (PMC4859512; doi:10.1371/journal.pone.0154490)
Supplement: S3 Fig — The membrane potential disruption was followed by monitoring Disc3(5) fluorescence. (PDF) [file pone.0154490.s003.pdf]

Inner membrane depolarization assay

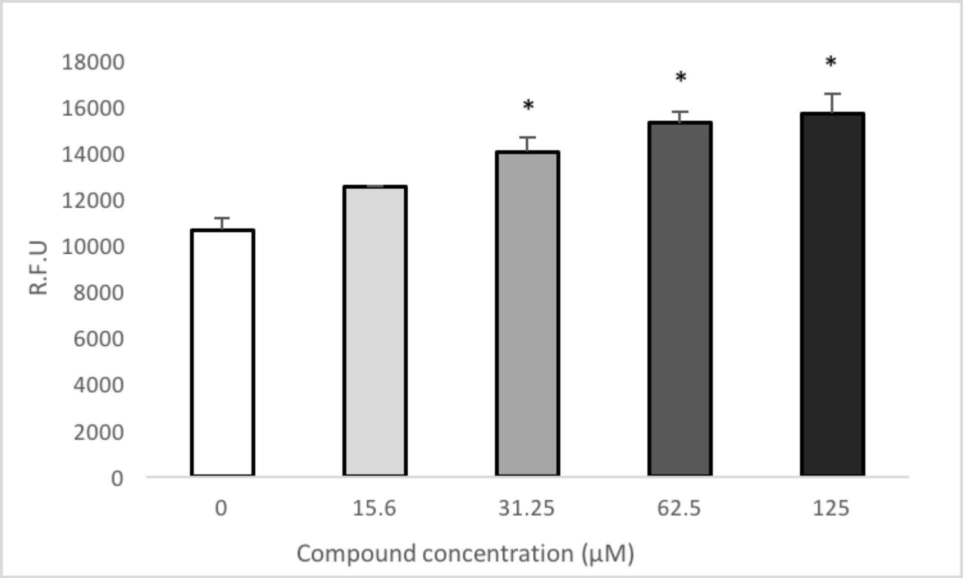

An overnight culture of PA01 was diluted 100 fold in MHII broth. Cells were grown to a mid-logarithmic phase, recovered by centrifugation (3000 X g for 20 min) and washed in 5 mM 4-(2-hydroxyethyl)piperazine-1-ethanesulfonic acid (HEPES, pH 7.0) containing 10 mM EDTA. After 5 min incubation at room temperature, cells were recovered by centrifugation (3000 X g for 20 min), and resuspended in 5 mM HEPES (pH 7.0) containing 50 mM glucose, OD600 nm was adjusted to 0.29. Five µl of a 160 µM suspension of [3,3'-Dipropylthiadicarbocyanine iodide] (Disc$_3$(5)) were added after 90 sec to 100 µl of a bacterial suspension, in a 96-well Greiner black microplate, to reach 8 µM final concentration. After a 16.5 min incubation period allowing incorporation of the dye into the polarized membrane, 10 µl of the appropriate compound concentration were added. Fluorescence was monitored every 30 sec on an Infinite M200 microplate reader (Tecan; excitation wavelength 622nm and emission wavelength 690nm). Assays were performed in two independent experiments and statistically significance was assessed using a student's t-test. A p-value ≤ 0.05 was considered significant.
