# Supplementary material for: Polyamino-Isoprenic Derivatives Block Intrinsic Resistance of P. aeruginosa to Doxycycline and Chloramphenicol In Vitro
Source: PLoS One. 2016 May 6;11(5):e0154490. doi: 10.1371/journal.pone.0154490 (PMC4859512; doi:10.1371/journal.pone.0154490)
Supplement: S1 Table — The antibiotic resistance of each isolate for commonly used antibiotics and the MIC for doxycycline are indicated. (PDF) [file pone.0154490.s004.pdf]

Isolates	Sampling location	Sampling date	Patient age	Doxycycline MIC (mg/L)	Main antibiotic resistance
**PA2013-LAV01**	Superficial pus	13/11/2012	80	32	TIC, TCC, FOS, TET, MIN, TGC, CHL, CAR
**PA2013-LAV02**	Urine	12/11/2012	95	32	TIC, TCC, FOS, TET, MIN, TGC, CHL,
**PA2013-LAV03**	Urine	13/11/2012	20	>32	PIP, PIP/TZP, GEN, CAZ, TIC, TCC, TOB, IPM, CIP, AMK, LVX, OFX, TET, MIN, TGC, CHL, NET, CAR
**PA2013-LAV04**	Urine	14/11/2012	81	32	TIC, TCC, FOS, TET, MIN, TGC, CHL,
**PA2013-LAV05**	Lung	28/11/2012	81	>32	GEN, TIC, TCC, TOB, CIP, AMK, LVX, OFX, TET, MIN, TGC, CHL, NET, CAR
**PA2013-LAV06**	Lung	29/11/2012	70	32	FOS, OFX, TET, MIN, TGC, CHL, NET,
**PA2013-LAV07**	Superficial pus	01/12/2012	88	32	TIC, TCC, FOS, TET, MIN, TGC, CHL,
**PA2013-LAV08**	Bone biopsy	30/11/2012	63	32	FOS, TET, MIN, TGC, CHL,
**PA2013-LAV09**	Lung	30/11/2012	62	>32	TCC, FOS, TET, MIN, TGC, CHL, NET,
**PA2013-LAV10**	Superficial pus	30/11/2012	74	32	TCC, FOS, TET, MIN, TGC, CHL,
**PA2013-LAV11**	Superficial pus	03/12/2012	83	32	TIC, TCC, FOS, TET, MIN, TGC, CHL,
**PA2013-LAV12**	Urine	02/12/2012	87	32	TIC, TCC, FOS, TET, MIN, TGC, CHL,
**PA2013-LAV13**	Superficial pus	10/12/2012	80	32	FOS, TET, MIN, TGC, CHL, STR, CAR
**PA2013-LAV14**	Urine	10/12/2012	85	>32	GEN, TIC, TCC, TOB, CIP, LVX, OFX, TET, MIN, TGC, CHL, NET, CAR
**PA2013-LAV15**	Bone biopsy	14/12/2012	92	>32	FOS, TET, MIN, TGC, CHL,
**PA2013-LAV16**	Catheter	13/12/2012	40	32	FOS, TET, MIN, TGC, CHL,
**PA2013-LAV17**	Redon drain	15/12/2012	81	32	PIP, PIP/TZP, AZT, CAZ, TIC, TCC, FEP, FOS, TET, MIN, TGC, CHL, CAR
**PA2013-LAV18**	Ear	14/12/2012	40	>32	FOS, TET, MIN, TGC, CHL,
**PA2013-LAV19**	Stool sample	16/12/2012	66	32	PIP, PIP/TZP, CAZ, TIC, TCC, IPM, MEM, FOS, TET, MIN, TGC, CHL, CAR
**PA2013-LAV20**	Genital	17/12/2012	62	>32	GEN, IPM, FOS, TET, MIN, TGC, CHL, NET,

TIC : ticarcillin, TCC : ticarcillin/clavulanic acid, FOS : fosfomycin, TET : tetracycline, MIN, minocycline, TCG, tigecycline, CAR : carbenicillin, PIP : piperacillin, PIP/TZP : piperacillin/tazobactam, GEN : gentamycin, CAZ : ceftazidime, FEP : cefepime, TOB : tobramycin, IPM : imipenem, CIP : ciprofloxacin, AMK : amikacin, LVX : levofloxacin, OFX : ofloxacin, NET : netilmicin, STR, streptomycin, MEM : meropenem.
