# Supplementary material for: Polyamino-Isoprenic Derivatives Block Intrinsic Resistance of P. aeruginosa to Doxycycline and Chloramphenicol In Vitro
Source: PLoS One. 2016 May 6;11(5):e0154490. doi: 10.1371/journal.pone.0154490 (PMC4859512; doi:10.1371/journal.pone.0154490)
Supplement: S2 Table — For each concentration of the combination between, (a) doxycycline and compound 3; (b) doxycycline and PAßN, the FIC index is indicated. (PDF) [file pone.0154490.s005.pdf]

Doxycycline		Concentration of compound **3** associated (µM)	FIC index	Effect
MIC (µg/ml)	MIC associated with compound 3 (µg/ml)			
32	16	0.18	**0.50**	Synergistic
	8	0.73	**0.26**	Synergistic
	4	2.93	**0.16**	Synergistic
	2	2.93	**0.09**	Synergistic
	1	5.86	**0.09**	Synergistic
	0.5	11.72	**0.14**	Synergistic
	0.25	23.44	**0.26**	Synergistic
	0.125	46.88	**0.50**	Synergistic
	0.0625	93.75	**1**	Additive

a)

Doxycycline		Concentration of PAßN associated (µM)	FIC index	Effect
MIC (µg/ml)	MIC associated with PAßN (µg/ml)			
32	16	2	**0.5**	Synergistic
	8	4	**0.25**	Synergistic
	4	8	**0.13**	Synergistic
	2	32	**0.09**	Synergistic
	1	32	**0.06**	Synergistic
	0.5	32	**0.05**	Synergistic
	0.25	32	**0.04**	Synergistic
	0.125	128	**0.13**	Synergistic
	0.0625	256	**0.25**	Additive

b)
