# Supplementary material for: Polyamino-Isoprenic Derivatives Block Intrinsic Resistance of P. aeruginosa to Doxycycline and Chloramphenicol In Vitro
Source: PLoS One. 2016 May 6;11(5):e0154490. doi: 10.1371/journal.pone.0154490 (PMC4859512; doi:10.1371/journal.pone.0154490)
Supplement: S3 Table — (PDF) [file pone.0154490.s006.pdf]

		MIC (mg/L)			
Strains	Compounds	Ceftazidime	Ticarcillin	Ciprofloxacin	Amikacin
PA01	∅	2	32-64	0.125	8
	**3** 10µM	2	16	0.125	64
	PA***β***N 10µM	2	32	0.125	16
	PA***β***N 30µM	1	32	≦0.06	16
PT629	∅	8	>64	0.5-1	4
	**3** 10µM	8	64	0.5	64
	PA***β***N 10µM	8	>64	0.5	4
	PA***β***N 30µM	2-4	>64	0.5	4-8
PA01 ERY^R	∅	2	32-64	0.125	1
	**3** 10µM	2	16	0.125	32
	PA***β***N 10µM	2	32	0.125	2
	PA***β***N 30µM	2	32	≦0.06	2
PA0-7H	∅	1	8	4	4
	**3** 10µM	1	4	1	64
	PA***β***N 10µM	1	8	4	4
	PA***β***N 30µM	1	8	4	4
CMZ091	∅	2	16-32	0.5	32
	**3** 10µM	2	8	0.25	>64
	PA***β***N 10µM	2	16-32	0.5	64
	PA***β***N 30µM	2	16-32	0.25	32-64
